# Supplementary material for: Metabolomic profiles in Jamaican children with and without autism spectrum disorder
Source: ArXiv. 2024 Mar 11:arXiv:2403.07147v1. Preprint. [Version 1] (PMC10980079)
Supplement: 1 [file NIHPP2403.07147V1-supplement-1.pdf]

# Supplement

**Metabolomic profiles in Jamaican children with and without autism spectrum disorder**

**Figure S1. Batch effect correction.**

**A.** Data representation using first two principal components before the correction.

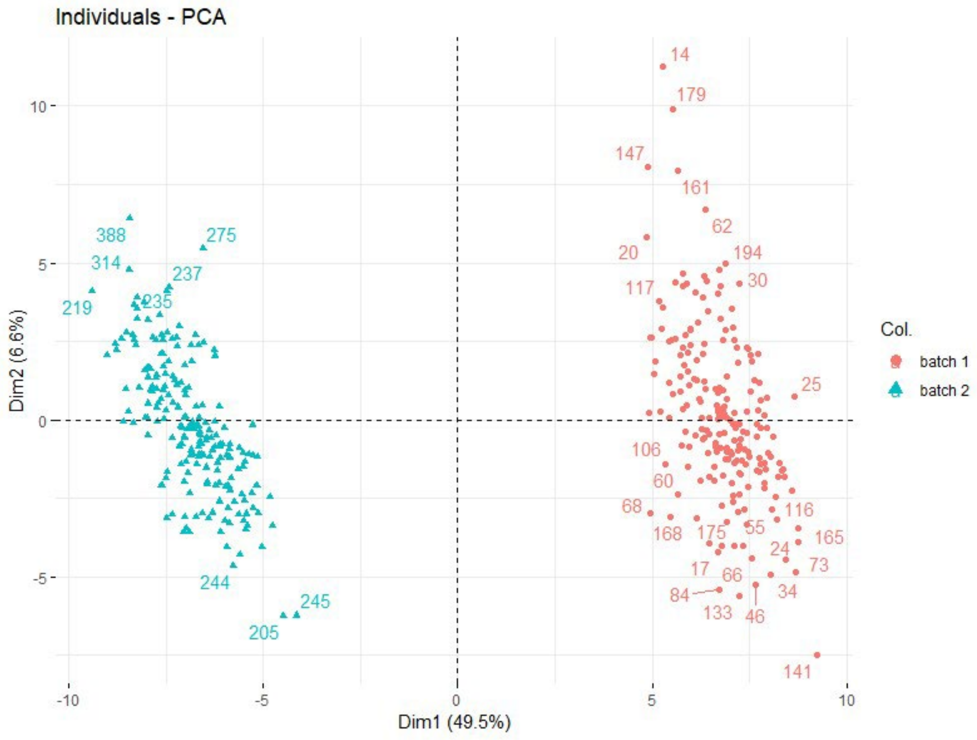

**B.** Data representation using first two principal components after the correction.

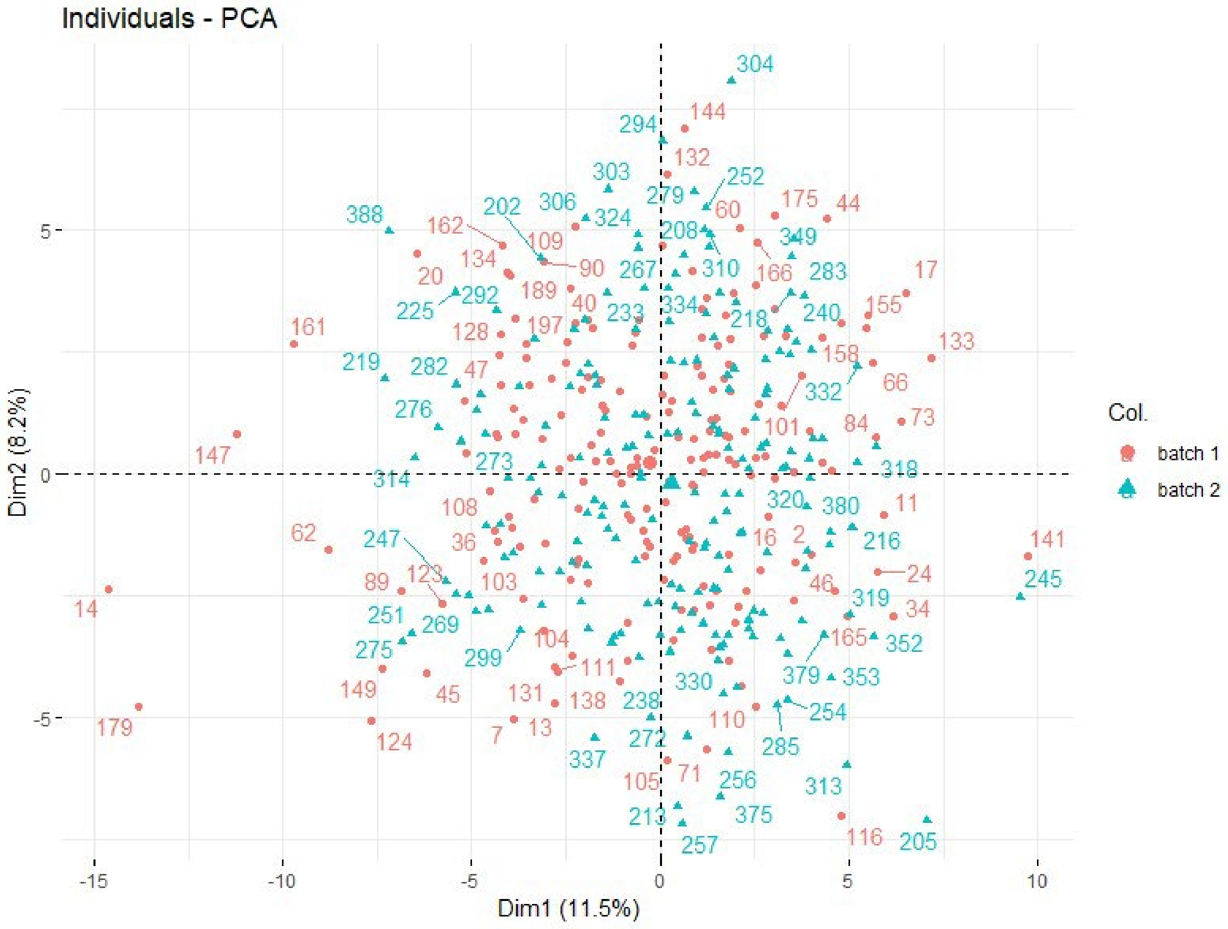

**Figure S2. Quality assessment.**

**A.** Metabolite clustering for the TD children group (controls).

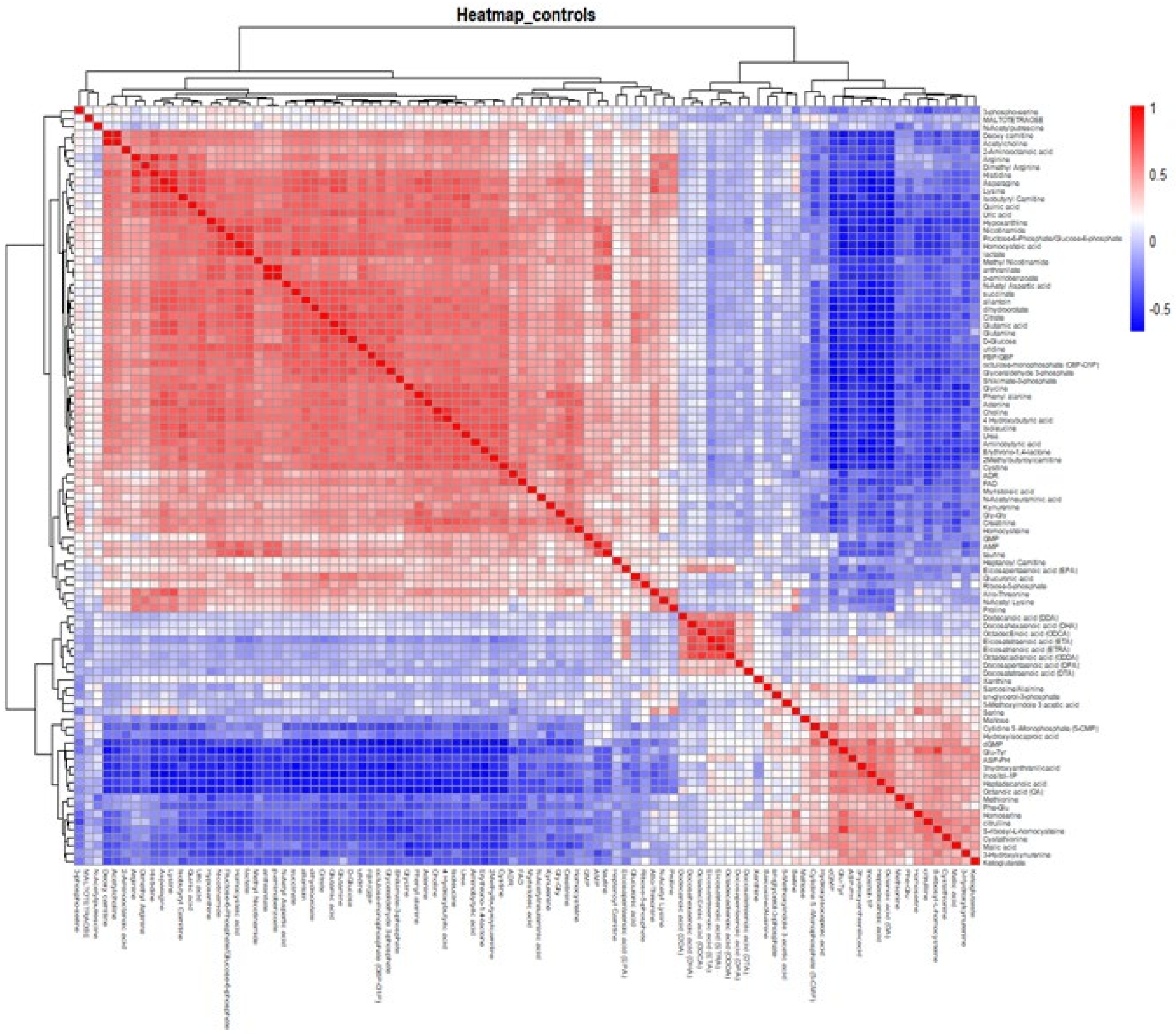

**B.** Metabolite clustering for the ASD group (cases).

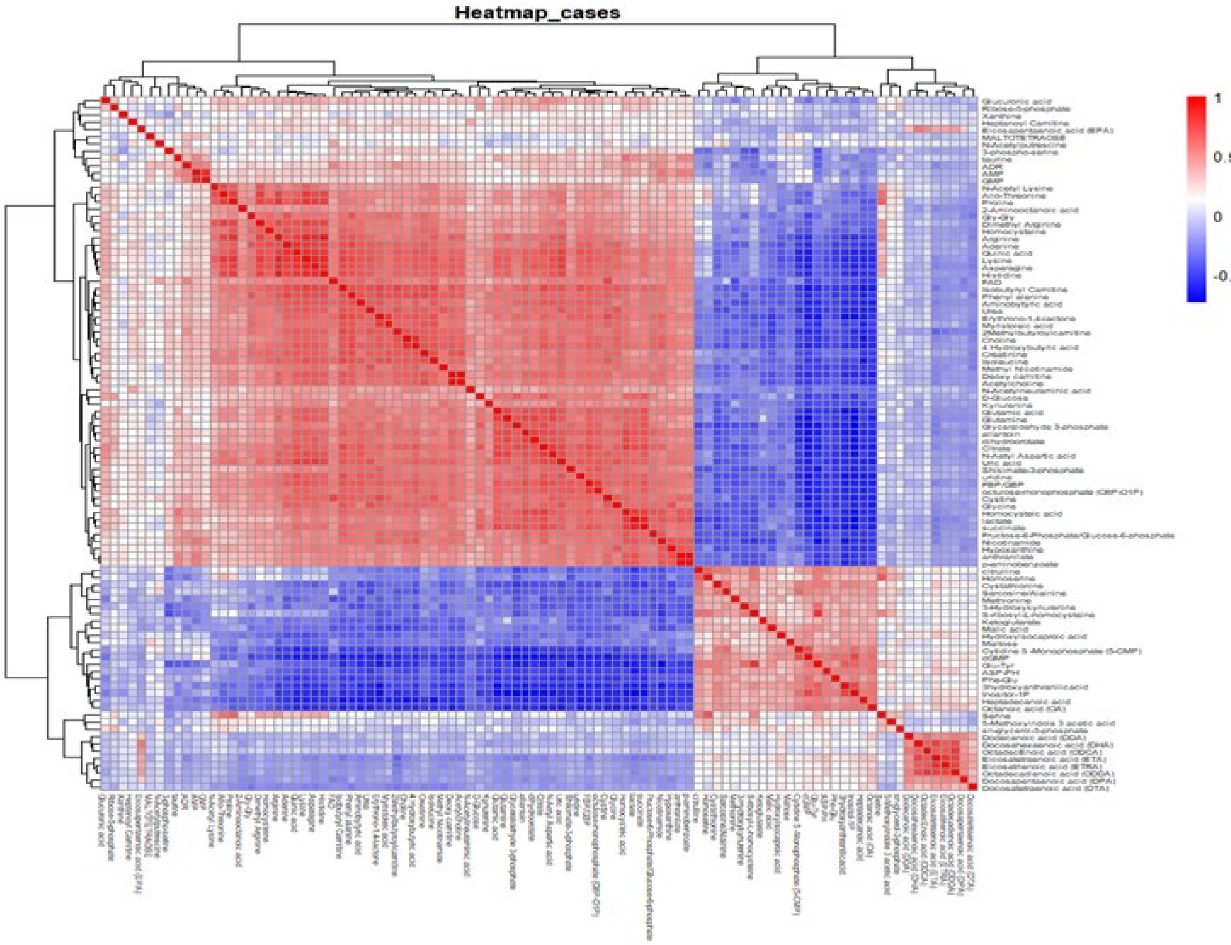

**Table S1.** By performing the Chi-square test or Fisher's exact test (*), we investigated the potential distinctions in dietary patterns between ASD and TD control groups based on food consumption scores obtained from questionnaires.

| | TD control group (N=200) | ASD group (N=200) | Total (N=400) | *p*-value |
|---|---|---|---|---|
| **Sea fish** | | | | 0.004 |
| Consumed | 133 (66.5%) | 105 (52.5%) | 238 (59.5%) | |
| Not Consumed | 67 (33.5%) | 95 (47.5%) | 162 (40.5%) | |
| **Fresh water** | | | | 0.182 |
| Consumed | 49 (24.5%) | 38 (19.0%) | 87 (21.8%) | |
| Not Consumed | 151 (75.5%) | 162 (81.0%) | 313 (78.2%) | |
| **Sardine** | | | | < 0.001 |
| Consumed | 170 (85.0%) | 139 (69.5%) | 309 (77.2%) | |
| Not Consumed | 30 (15.0%) | 61 (30.5%) | 91 (22.8%) | |
| **Tuna** | | | | 0.196 |
| Consumed | 69 (34.5%) | 57 (28.5%) | 126 (31.5%) | |
| Not Consumed | 131 (65.5%) | 143 (71.5%) | 274 (68.5%) | |
| **Saltfish** | | | | < 0.001 |
| Consumed | 158 (79.0%) | 113 (56.5%) | 271 (67.8%) | |
| Not Consumed | 42 (21.0%) | 87 (43.5%) | 129 (32.2%) | |
| **Shell fish lobster** | | | | < 0.001 |
| Consumed | 33 (16.5%) | 6 (3.0%) | 39 (9.8%) | |
| Not Consumed | 167 (83.5%) | 194 (97.0%) | 361 (90.2%) | |
| **Shrimp** | | | | < 0.001 |
| Consumed | 33 (16.5%) | 6 (3.0%) | 39 (9.8%) | |
| Not Consumed | 167 (83.5%) | 194 (97.0%) | 361 (90.2%) | |
| **Packaged fish** | | | | 0.388 |
| Consumed | 38 (19.0%) | 45 (22.5%) | 83 (20.8%) | |
| Not Consumed | 162 (81.0%) | 155 (77.5%) | 317 (79.2%) | |
| **Beef** | | | | 0.085 |
| Consumed | 92 (46.0%) | 75 (37.5%) | 167 (41.8%) | |
| Not Consumed | 108 (54.0%) | 125 (62.5%) | 233 (58.2%) | |
| **Lamb mutton** | | | | 0.110 |
| Consumed | 33 (16.5%) | 22 (11.0%) | 55 (13.8%) | |
| Not Consumed | 167 (83.5%) | 178 (89.0%) | 345 (86.2%) | |
| **Goat** | | | | < 0.001 |
| Consumed | 101 (50.5%) | 68 (34.0%) | 169 (42.2%) | |

| | TD control group (N=200) | ASD group (N=200) | Total (N=400) | *p*-value |
|---|---|---|---|---|
| Not Consumed | 99 (49.5%) | 132 (66.0%) | 231 (57.8%) | |
| **Pork** | | | | < 0.001 |
| Consumed | 112 (56.0%) | 79 (39.5%) | 191 (47.8%) | |
| Not Consumed | 88 (44.0%) | 121 (60.5%) | 209 (52.2%) | |
| **Liver** | | | | 0.009 |
| Consumed | 117 (58.5%) | 91 (45.5%) | 208 (52.0%) | |
| Not Consumed | 83 (41.5%) | 109 (54.5%) | 192 (48.0%) | |
| **Chicken** | | | | 0.006* |
| Consumed | 196 (98.0%) | 184 (92.0%) | 380 (95.0%) | |
| Not Consumed | 4 (2.0%) | 16 (8.0%) | 20 (5.0%) | |
| **Milk** | | | | 0.229 |
| Consumed | 98 (49.0%) | 86 (43.0%) | 184 (46.0%) | |
| Not Consumed | 102 (51.0%) | 114 (57.0%) | 216 (54.0%) | |
| **Cheese** | | | | < 0.001 |
| Consumed | 165 (82.5%) | 134 (67.0%) | 299 (74.8%) | |
| Not Consumed | 35 (17.5%) | 66 (33.0%) | 101 (25.2%) | |
| **Yogurt** | | | | 0.004 |
| Consumed | 68 (34.0%) | 42 (21.0%) | 110 (27.5%) | |
| Not Consumed | 132 (66.0%) | 158 (79.0%) | 290 (72.5%) | |
| **Eggs** | | | | < 0.001 |
| Consumed | 172 (86.0%) | 144 (72.0%) | 316 (79.0%) | |
| Not Consumed | 28 (14.0%) | 56 (28.0%) | 84 (21.0%) | |
| **Rice** | | | | < 0.001* |
| Consumed | 199 (99.5%) | 178 (89.0%) | 377 (94.2%) | |
| Not Consumed | 1 (0.5%) | 22 (11.0%) | 23 (5.8%) | |
| **Fried dumpling** | | | | 0.140 |
| Consumed | 154 (77.0%) | 141 (70.5%) | 295 (73.8%) | |
| Not Consumed | 46 (23.0%) | 59 (29.5%) | 105 (26.2%) | |
| **Boiled dumpling** | | | | < 0.001 |
| Consumed | 184 (92.0%) | 154 (77.0%) | 338 (84.5%) | |
| Not Consumed | 16 (8.0%) | 46 (23.0%) | 62 (15.5%) | |
| **White bread** | | | | < 0.001 |
| Consumed | 140 (70.0%) | 103 (51.5%) | 243 (60.8%) | |
| Not Consumed | 60 (30.0%) | 97 (48.5%) | 157 (39.2%) | |
| **Whole wheat bread** | | | | 0.760 |
| Consumed | 121 (60.5%) | 118 (59.0%) | 239 (59.8%) | |
| Not Consumed | 79 (39.5%) | 82 (41.0%) | 161 (40.2%) | |

| | TD control group (N=200) | ASD group (N=200) | Total (N=400) | *p*-value |
|---|---|---|---|---|
| **Cakes bun** | | | | 0.003 |
| Consumed | 164 (82.0%) | 138 (69.0%) | 302 (75.5%) | |
| Not Consumed | 36 (18.0%) | 62 (31.0%) | 98 (24.5%) | |
| **Porridge** | | | | 0.003 |
| Consumed | 184 (92.0%) | 164 (82.0%) | 348 (87.0%) | |
| Not Consumed | 16 (8.0%) | 36 (18.0%) | 52 (13.0%) | |
| **Cold cereal** | | | | < 0.001 |
| Consumed | 157 (78.5%) | 116 (58.0%) | 273 (68.2%) | |
| Not Consumed | 43 (21.5%) | 84 (42.0%) | 127 (31.8%) | |
| **Macaroni** | | | | < 0.001 |
| Consumed | 179 (89.5%) | 150 (75.0%) | 329 (82.2%) | |
| Not Consumed | 21 (10.5%) | 50 (25.0%) | 71 (17.8%) | |
| **Peas** | | | | < 0.001 |
| Consumed | 146 (73.0%) | 106 (53.0%) | 252 (63.0%) | |
| Not Consumed | 54 (27.0%) | 94 (47.0%) | 148 (37.0%) | |
| **Beans** | | | | 0.011 |
| Consumed | 94 (47.0%) | 69 (34.5%) | 163 (40.8%) | |
| Not Consumed | 106 (53.0%) | 131 (65.5%) | 237 (59.2%) | |
| **Nuts** | | | | < 0.001 |
| Consumed | 158 (79.0%) | 95 (47.5%) | 253 (63.2%) | |
| Not Consumed | 42 (21.0%) | 105 (52.5%) | 147 (36.8%) | |
| **Yam** | | | | 0.008 |
| Consumed | 147 (73.5%) | 122 (61.0%) | 269 (67.2%) | |
| Not Consumed | 53 (26.5%) | 78 (39.0%) | 131 (32.8%) | |
| **Carrot** | | | | < 0.001 |
| Consumed | 174 (87.0%) | 147 (73.5%) | 321 (80.2%) | |
| Not Consumed | 26 (13.0%) | 53 (26.5%) | 79 (19.8%) | |
| **Lettuce** | | | | < 0.001 |
| Consumed | 107 (53.5%) | 57 (28.5%) | 164 (41.0%) | |
| Not Consumed | 93 (46.5%) | 143 (71.5%) | 236 (59.0%) | |
| **Callaloo** | | | | 0.001 |
| Consumed | 153 (76.5%) | 123 (61.5%) | 276 (69.0%) | |
| Not Consumed | 47 (23.5%) | 77 (38.5%) | 124 (31.0%) | |
| **Cabbage** | | | | < 0.001 |
| Consumed | 148 (74.0%) | 109 (54.5%) | 257 (64.2%) | |
| Not Consumed | 52 (26.0%) | 91 (45.5%) | 143 (35.8%) | |
| **String beans** | | | | 0.020 |

| | TD control group (N=200) | ASD group (N=200) | Total (N=400) | *p*-value |
|---|---|---|---|---|
| Consumed | 59 (29.5%) | 39 (19.5%) | 98 (24.5%) | |
| Not Consumed | 141 (70.5%) | 161 (80.5%) | 302 (75.5%) | |
| **Tomatoes** | | | | < 0.001 |
| Consumed | 142 (71.0%) | 105 (52.5%) | 247 (61.8%) | |
| Not Consumed | 58 (29.0%) | 95 (47.5%) | 153 (38.2%) | |
| **Ackee** | | | | < 0.001 |
| Consumed | 155 (77.5%) | 94 (47.0%) | 249 (62.2%) | |
| Not Consumed | 45 (22.5%) | 106 (53.0%) | 151 (37.8%) | |
| **Avocado** | | | | < 0.001 |
| Consumed | 106 (53.0%) | 42 (21.0%) | 148 (37.0%) | |
| Not Consumed | 94 (47.0%) | 158 (79.0%) | 252 (63.0%) | |
| **Green banana** | | | | < 0.001 |
| Consumed | 155 (77.5%) | 123 (61.5%) | 278 (69.5%) | |
| Not Consumed | 45 (22.5%) | 77 (38.5%) | 122 (30.5%) | |
| **Fried plantain** | | | | < 0.001 |
| Consumed | 173 (86.5%) | 142 (71.0%) | 315 (78.8%) | |
| Not Consumed | 27 (13.5%) | 58 (29.0%) | 85 (21.2%) | |
| **Ripe banana** | | | | < 0.001 |
| Consumed | 189 (94.5%) | 162 (81.0%) | 351 (87.8%) | |
| Not Consumed | 11 (5.5%) | 38 (19.0%) | 49 (12.2%) | |
| **Oranges** | | | | < 0.001 |
| Consumed | 181 (90.5%) | 134 (67.0%) | 315 (78.8%) | |
| Not Consumed | 19 (9.5%) | 66 (33.0%) | 85 (21.2%) | |
| **Tangerine** | | | | < 0.001 |
| Consumed | 95 (47.5%) | 52 (26.0%) | 147 (36.8%) | |
| Not Consumed | 105 (52.5%) | 148 (74.0%) | 253 (63.2%) | |
| **Grapes** | | | | < 0.001 |
| Consumed | 123 (61.5%) | 79 (39.5%) | 202 (50.5%) | |
| Not Consumed | 77 (38.5%) | 121 (60.5%) | 198 (49.5%) | |
| **Otaheite apples** | | | | < 0.001 |
| Consumed | 169 (84.5%) | 128 (64.0%) | 297 (74.2%) | |
| Not Consumed | 31 (15.5%) | 72 (36.0%) | 103 (25.8%) | |
| **Pineapples** | | | | < 0.001 |
| Consumed | 143 (71.5%) | 90 (45.0%) | 233 (58.2%) | |
| Not Consumed | 57 (28.5%) | 110 (55.0%) | 167 (41.8%) | |
| **American apples** | | | | 0.002 |
| Consumed | 78 (39.0%) | 49 (24.5%) | 127 (31.8%) | |

| | TD control group (N=200) | ASD group (N=200) | Total (N=400) | *p*-value |
|---|---|---|---|---|
| Not Consumed | 122 (61.0%) | 151 (75.5%) | 273 (68.2%) | |
| **Guinep** | | | | 0.041 |
| Consumed | 12 (6.0%) | 4 (2.0%) | 16 (4.0%) | |
| Not Consumed | 188 (94.0%) | 196 (98.0%) | 384 (96.0%) | |
| **Peach** | | | | 0.041* |
| Consumed | 12 (6.0%) | 4 (2.0%) | 16 (4.0%) | |
| Not Consumed | 188 (94.0%) | 196 (98.0%) | 384 (96.0%) | |
| **Plums** | | | | < 0.001 |
| Consumed | 98 (49.0%) | 24 (12.0%) | 122 (30.5%) | |
| Not Consumed | 102 (51.0%) | 176 (88.0%) | 278 (69.5%) | |
| **Strawberry** | | | | 0.388 |
| Consumed | 21 (10.5%) | 16 (8.0%) | 37 (9.2%) | |
| Not Consumed | 179 (89.5%) | 184 (92.0%) | 363 (90.8%) | |
| **Naseberry** | | | | 0.004 |
| Consumed | 70 (35.0%) | 44 (22.0%) | 114 (28.5%) | |
| Not Consumed | 130 (65.0%) | 156 (78.0%) | 286 (71.5%) | |
| **Sweetsop** | | | | < 0.001 |
| Consumed | 69 (34.5%) | 23 (11.5%) | 92 (23.0%) | |
| Not Consumed | 131 (65.5%) | 177 (88.5%) | 308 (77.0%) | |
| **Mango** | | | | < 0.001 |
| Consumed | 186 (93.0%) | 148 (74.0%) | 334 (83.5%) | |
| Not Consumed | 14 (7.0%) | 52 (26.0%) | 66 (16.5%) | |
| **June plum** | | | | < 0.001 |
| Consumed | 142 (71.0%) | 74 (37.0%) | 216 (54.0%) | |
| Not Consumed | 58 (29.0%) | 126 (63.0%) | 184 (46.0%) | |
| **Juices** | | | | < 0.001 |
| Consumed | 186 (93.0%) | 161 (80.5%) | 347 (86.8%) | |
| Not Consumed | 14 (7.0%) | 39 (19.5%) | 53 (13.2%) | |
| **Beverages** | | | | 0.514 |
| Consumed | 181 (90.5%) | 177 (88.5%) | 358 (89.5%) | |
| Not Consumed | 19 (9.5%) | 23 (11.5%) | 42 (10.5%) | |
| **Soft drinks** | | | | 0.057 |
| Consumed | 104 (52.0%) | 85 (42.5%) | 189 (47.2%) | |
| Not Consumed | 96 (48.0%) | 115 (57.5%) | 211 (52.8%) | |
| **Tea substitutes** | | | | < 0.001 |
| Consumed | 185 (92.5%) | 160 (80.0%) | 345 (86.2%) | |
| Not Consumed | 15 (7.5%) | 40 (20.0%) | 55 (13.8%) | |

| | TD control group (N=200) | ASD group (N=200) | Total (N=400) | *p*-value |
|---|---|---|---|---|
| **Canned food** | | | | < 0.001 |
| Consumed | 162 (81.0%) | 122 (61.0%) | 284 (71.0%) | |
| Not Consumed | 38 (19.0%) | 78 (39.0%) | 116 (29.0%) | |
| **Aluminum foil** | | | | 0.303 |
| Consumed | 41 (20.5%) | 33 (16.5%) | 74 (18.5%) | |
| Not Consumed | 159 (79.5%) | 167 (83.5%) | 326 (81.5%) | |
| **Unpeeled fruits** | | | | < 0.001 |
| Consumed | 179 (89.5%) | 124 (62.0%) | 303 (75.8%) | |
| Not Consumed | 21 (10.5%) | 76 (38.0%) | 97 (24.2%) | |
| **Animal fat** | | | | 0.003 |
| Consumed | 44 (22.0%) | 22 (11.0%) | 66 (16.5%) | |
| Not Consumed | 156 (78.0%) | 178 (89.0%) | 334 (83.5%) | 0.004 |
